# Editorial: Special issue imaging and pain

**DOI:** 10.1016/j.ostima.2026.100392

**Published:** 2026-02-13

**Authors:** Mohamed Jarraya, Mylène P Jansen

**Affiliations:** a Massachusetts General Hospital, Harvard Medical School, Boston, United States; b Department of Rheumatology & Clinical Immunology, University Medical Center Utrecht, the Netherlands

This special issue of *Osteoarthritis Imaging* commemorates the 19th International Workshop of Osteoarthritis Imaging (IWOAI), held in Marrakech, Morocco between June 25th and 28th, 2024. The contributions gathered in this issue are based on the key presentations of the invited speakers of the IWOAI 2024 and reflect the multifaceted and evolving understanding of osteoarthritis (OA)-related pain and its associations with structural biomarkers. Collectively, they emphasize the need for improved phenotyping of pain, consideration of central sensitization, refined anatomical and morphological stratification, and rigorous validation of imaging-based endpoints.

Pain mechanisms in OA are heterogeneous, with important implications for both research and clinical care. In their perspective on *deconstructing types of osteoarthritis*, Hunter and Deveza argue that meaningful progress in therapy development depends not only on the identification of robust biomarkers and clinically relevant endpoints, but also on improved phenotyping of pain and clear definition of OA pain subtypes [1]. Consideration of central sensitization is particularly critical in this context, as altered central pain processing contributes to the frequently observed weak association between structural changes and pain outcomes. Walsh et al. emphasize the need to account for central sensitization in clinical trials of disease-modifying OA drugs, since heightened central pain processing may mask potential analgesic benefits arising from structural modification [2]. In addition to pain subtypes and phenotypes, structural morphotypes represent another path for more personalized treatment strategies. Chin and Collins discuss the promise of unsupervised clustering methods to uncover patient subgroups based on anatomical and morphological features, while critically examining the analytic challenges inherent to these approaches [3].

This issue also discusses composite imaging outcomes in OA. Composite measures are widely used in rheumatic and musculoskeletal diseases to evaluate outcomes of treatment or risk factors for disease [4,5]. Composite measures have several advantages including a potential increase in statistical power. The best-known example in medicine is the MACE score for cardiovascular outcomes [6]. Original research by McGinley et al. assesses multiple measures of patellofemoral dysplasia against both single-structure and composite MRI outcomes over two years [7]. For patellofemoral dysplasia, the authors show aggregating structural features may dilute rather than enhance signal, underscoring the need for careful validation of composite outcomes before their adoption. In addition, Eckstein et al. discuss, in a narrative format, the vast array of options for multi-component/composite endpoints in OA, calling on careful validation tailored to both, a drug’s unique mechanism of action, and to the structural morphotype [8].

Among the available imaging biomarkers, contrast-enhanced (CE) MRI–detected synovitis represents a key marker of synovial inflammation. Berenbaum highlights the potential of CE-MRI as a surrogate indicator of synovial health, enabling precise visualization of inflammatory activity and its relationship to both disease progression and pain [9]. Beyond synovitis, subchondral bone is another important tissue of OA pathophysiology including disease onset and structural progression. Mobasheri et al. propose a mechano-inflammatory disruption of the bone marrow microenvironment as a central mechanism linking structural progression and pain [10]. Complementing this perspective, Moradi et al. synthesize current evidence on bone marrow lesions as key imaging biomarkers in knee OA, highlighting their detection, prognostic significance, and therapeutic relevance [11]. Finally, Omoumi discusses the complementary roles of MRI and CT in the assessment of cartilage and meniscal pathology in OA [12].

Two contributions address genicular artery embolization (GAE) and genicular nerve ablation (GNA) in the management of refractory pain in knee OA. Shami et al. outline the technical principles of each approach, targeting synovial hypervascularity and sensory nerve input, respectively, and review current evidence demonstrating short- to mid-term improvements in pain and function with favorable safety profiles [13]. Although early uncontrolled case series suggested substantial and durable improvements in pain and function, the absence of appropriate control groups has limited interpretability. Importantly, evidence from sham-controlled randomized trials has been inconsistent, with the two most recent studies failing to demonstrate superiority over placebo [14,15]. van Zadelhoff et al. underscores the need for caution, highlighting the gap between biological plausibility, early enthusiasm, and robust evidence [16].

Finally, artificial intelligence (AI)–driven approaches in OA research have been the subject of rapidly growing enthusiasm. In seeking to clarify the potential role of AI in addressing the long-standing disconnect between structure and pain, a narrative review by Bayramoglu et al. provides a timely and necessary reality check [17]. The authors critically examine why the promise of machine learning and deep learning has yet to translate into routine clinical relevance, highlighting fundamental challenges such as limited longitudinal data available for training, small and imbalanced cohorts, difficulties in aligning multimodal input over time, and pain assessment tools that inadequately capture the multidimensional nature of symptoms (such as psychosocial and demographic factors).

Together, the articles in this special issue highlight the rapid evolution of OA imaging and its central role in refining pain phenotypes, validating imaging biomarkers, and strengthening trial endpoints. We are grateful to the authors for their excellent contributions and to the reviewers and entire editorial team for their support, making this a most valuable contribution to the community. We hope this issue will inform future research and accelerate progress toward more precise, mechanism-informed, and patient-centered OA therapies.

## Declaration of competing interest

The authors declare that they have no known competing financial interests or personal relationships that could have appeared to influence the work reported in this paper.
